# Individuality manifests in the dynamic reconfiguration of large-scale brain networks during movie viewing

**DOI:** 10.1038/srep41414

**Published:** 2017-01-23

**Authors:** Changwon Jang, Elizabeth Quattrocki Knight, Chongwon Pae, Bumhee Park, Shin-Ae Yoon, Hae-Jeong Park

**Affiliations:** 1 BK21 PLUS Project for Medical Science, Yonsei University College of Medicine, Seoul, Republic of Korea; 2 Department of Nuclear Medicine, Department of Radiology, Department of Psychiatry, Severance Hospital, Yonsei University College of Medicine, Seoul, Republic of Korea; 3 Department of Psychiatry, Harvard Medical School, McLean Hospital, 115 Mill Street Belmont, MA 02478, USA; 4 Department of Statistics, Hankuk University of Foreign Studies, Yong-In, Republic of Korea; 5 Department of Cognitive Science, Yonsei University, Seoul, Republic of Korea

## Abstract

Individuality, the uniqueness that distinguishes one person from another, may manifest as diverse rearrangements of functional connectivity during heterogeneous cognitive demands; yet, the neurobiological substrates of individuality, reflected in inter-individual variations of large-scale functional connectivity, have not been fully evidenced. Accordingly, we explored inter-individual variations of functional connectivity dynamics, subnetwork patterns and modular architecture while subjects watched identical video clips designed to induce different arousal levels. How inter-individual variations are manifested in the functional brain networks was examined with respect to four contrasting divisions: edges within the anterior *versus* posterior part of the brain, edges with *versus* without corresponding anatomically-defined structural pathways, inter- *versus* intra-module connections, and rich club edge types. Inter-subject variation in dynamic functional connectivity occurred to a greater degree within edges localized to anterior rather than posterior brain regions, without adhering to structural connectivity, between modules as opposed to within modules, and in weak-tie local edges rather than strong-tie rich-club edges. Arousal level significantly modulates inter-subject variability in functional connectivity, edge patterns, and modularity, and particularly enhances the synchrony of rich-club edges. These results imply that individuality resides in the dynamic reconfiguration of large-scale brain networks in response to a stream of cognitive demands.

Individuality, or individual subjectivity, refers to a compilation of qualities that distinguish people from each other, not only in character and temperament, but also in the way they perceive, feel and perform a cognitive task. To date, individuality has been studied with regard to individual differences or variability in contrast to a common prototype or model. Human individual variability has been recognized at the behavioral level^1^,^2^ and with regard to brain activations during a specific type of cognitive performance^3^,^4^,^5^. Recent studies of human variability have focused on differences in “resting state” functional connectivity^6^,^7^. However, the neurobiological underpinnings of individuality, in response to a natural setting that demands diverse cognitive functions, remains to be investigated, particularly with respect to dynamic functional connectivity of brain network systems.

To date, some functional magnetic resonance imaging (fMRI) studies of inter-subject variability in the brain have explored the synchrony of regional brain “activity” among individuals while subjects perform the same task in a natural setting (mainly watching a movie)^8^,^9^,^10^,^11^,^12^,^13^. In these studies, neuronal synchrony was measured using inter-subject correlation (ISC; temporal) of regional brain activity to a series of stimuli. ISC has been used to determine whether the neuronal response in one individual’s brain is similar to the response in a separate individual’s brain while the subjects experience identical stimuli^9^. This approach detects which brain voxels show similar time courses (activity) across individuals (high inter-subject correlation) and which brain voxels show heterogeneous (and thus individualized) time courses during movie viewing. Therefore, ISC is considered to be a data-driven biomarker for localizing brain regions of high inter-individual similarity or variations. ISC of regional brain activity was consistently high in the sensory cortex; whereas, ISC was relatively low in the higher (or later) cognitive brain areas^9^,^14^,^15^,^16^, implying that higher cognitive brain regions encode individual differences.

Because brain regions do not operate in isolation, but function together to perform a task, identifying brain regions across subjects that respond synchronously to identical demands can only partially decipher the essence of brain individuality. Recently, the shift toward conceptually viewing the brain as a network system^17^,^18^ suggests that individual differences may exist in the orchestration of brain regions employed for a certain psychological process. Therefore, exploring various interactions among brain regions from the network perspective can better elucidate the neurobiological underpinnings of individual variability.

In this study, we investigated how inter-subject variability manifests in the large-scale brain network while individuals watched the same set of video clips. Unlike previous studies, which limited their examinations of inter-subject variability to assessing asynchrony of regional activation across individuals, we focused on inter-regional functional connectivity at each edge (edge strength) and patterns of edge sets (subnetwork architecture) involved in a perceptual task. In this paper, we use the term “perceptual,” to include not just the basic transmission of sensory signal information, but the emotional, cognitive, and attentional processing that gives rise to “understanding”.

We detailed inter-subject variability with respect to five aspects of functional connectivity: connectivity within the anterior *versus* posterior areas of the brain, existence of underlying structural connectivity, intra- *versus* inter-modular connectivity, connectivity types in reference to the rich club organization schema and interactions between these four contrasts and arousal levels.

Similar to previous studies^9^,^19^, we compared connectivity in the anterior brain areas, the regions generally responsible for higher order cognitions, to posterior brain areas, the structures essential for sensory/early level processing.

How structural brain (anatomic) networks correspond to functional connectivity has become an increasingly important framework for comprehending the brain^20^,^21^ and understanding of certain brain diseases^22^,^23^,^24^. In this respect, we investigated the effect of structural connectivity on inter-subject variability of functional connectivity.

The brain’s modular architecture segregates brain functions in a hierarchical manner^25^. To characterize brain modularity, researchers have proposed two slightly different schemas to describe modular structures; community arrangements^26^ and rich club organizations^27^. In a community structure, modules are defined as groups of densely interconnected nodes that are only sparsely linked with nodes residing in different modules^26^. Processing within a module, referred to as local integration, occurs primarily *via* strong short-ranged edges and creates an operational unit that performs a specialized function. Conversely, global integration in the community modular model refers to interactions between modules and most likely utilizes sparse, weak, but long-range edges. Community structures prioritize discrete operations over distributed local integration. Whereas, the “rich-club” organization emphasizes efficient information flow and thereby optimizes interactions or dense connections between modules in the network^28^. In the rich-club organization, a rich-club hub connects not only with many other feeder nodes (thus, composing a module via feeder edges), but also provides access to other rich-club hubs with dense rich-club edges. While intra-modular edges (or feeders) and rich-club edges may work as “strong ties”, edges not connected with any rich club nodes (referred to as local edges) may play as “weak ties”, similar to a framework established in social networks^29^. In this study, we assessed the inter-subject variability of weak versus strong ties, using the above structure as a framework, to explore the substrates that might account for individual variability.

Furthermore, we investigated how arousal levels differentially modulate inter-subject variability in connectivity, by considering the contribution of arousal to the contrasts described above, similar to the study of Nummenmaa, *et al*.^10^ that showed arousal effects on the voxel-wise inter-subject synchrony.

To test these hypotheses, we obtained fMRI images while participants watched a set of video clips designed to induce either low or high arousal levels. We evaluated the temporal synchronies of nodal activity and functional connectivity using temporal inter-subject correlations (ISC) of blood oxygenation level dependent signal (BOLD) changes. We also evaluated inter-subject similarity (ISS) for patterns of active edge sets within various modules (anterior/posterior, with/without corresponding structural connectivity, inter/intra modular, and rich club/feeder/local edges types) across individuals for each arousal category. In conclusion, we demonstrate that individuality resides in the dynamic reconfiguration of large-scale brain networks, modulated by arousal levels, in response to a stream of cognitive demands.

## Results

### Synchrony of nodal activity

**Figure 1:**
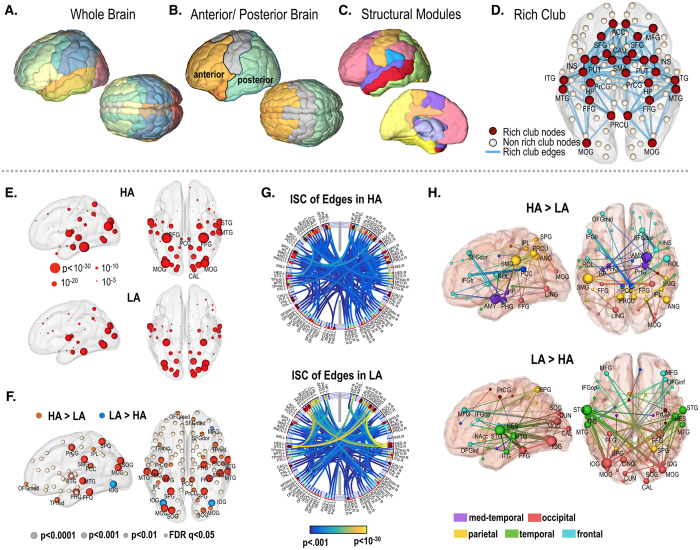
Subnetworks of the brain used in this study. Network node and edge definitions using the Automated Anatomical Labeling (AAL) map of the whole brain (**A**), anterior and posterior areas of the brain (**B**), modules defined by modularity optimization of structural networks for inter/intra-modular connectivity analysis (**C**) and rich-club nodes defined by structural networks (**D**). Rich-club nodes (node degree >16, red spheres) were found at the anterior cingulate cortex (ACC), caudate (CAU), fusiform gyrus (FFG), hippocampus (HP), inferior temporal gyrus (ITG), insula (INS), middle cingulate cortex (MCC), middle frontal gyrus (MFG), middle occipital gyrus (MOG), middle temporal gyrus (MTG), precentral gyrus (PrCG), precuneus (PRCU), putamen (PUT), superior dorsal frontal Gyrus (SFGdor) and supplementary motor area (SMA). Inter-subject correlation (ISC) of nodes and edges. (**E**) T-maps of ISC for nodal activity at high and low arousal states (HA, and LA) (one sample t-test). (**F**) Statistical difference of nodal synchronization (ISC) across individuals between the high and low arousal states (blue depicts greater synchrony in LA and orange represents greater synchrony in HA). FDR q < 0.05. (**G**) T-maps of ISC for dynamic functional connectivity in the high and low arousal state (one sample t-test). Edges with z-transformed ISC > 2 were displayed. (**H**) Statistical difference of edge synchronization (ISC of functional connectivity) across individuals between the high and low arousal states. FDR q < 0.05.

For nodes in the occipital, temporal and parietal lobes, the ISC was high; whereas, nodal ISC was low in the frontal lobe (Fig. 1E and F and Supplementary Table 1). High arousal was generally associated with a greater number of highly synchronous regions across subjects. In both hemispheres, the supra-marginal gyrus, superior occipital gyrus, posterior cingulate cortex, parahippocampal gyrus, middle temporal gyrus, precentral gyrus and the fusiform gyrus displayed higher ISC during the high arousal state than low arousal state. However, ISC in the bilateral inferior occipital gyri, was significantly higher during the low arousal condition (FDR < 0.05, Fig. 1F).

### Synchrony of edge dynamics

The ISCs of edges (functional connectivity) are displayed in Fig. 1G and H (Supplementary Table 2). The total number of synchronized edges (z value of the ISC > 1.96) and the average ISC for edges having a z value of greater than 1.96 are significantly higher in the high arousal state than in the low arousal state (p = 0.052 and p = 0.001, respectively).

High arousal tended to increase the ISC in those edges that interconnect high ISC nodes, such as the left supramarginal gyrus, right parahippocampal gyrus, right amygdala and bilateral precuneus (Fig. 1H). Whereas, the edges projecting from the visual and auditory areas were higher in the low arousal state than the high arousal state (FDR < 0.05) (Fig. 1H).

**Figure 2:**
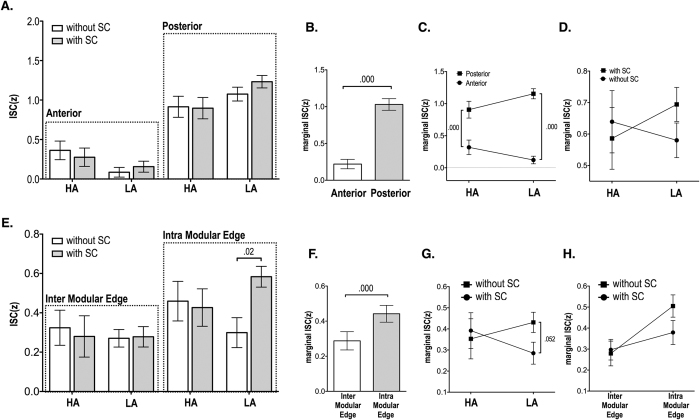
Statistical results for inter-subject correlation (ISC) of functional connectivity. (**A**) Three-way repeated measures ANOVA for arousal condition, anterior/posterior region condition and with/without structural connectivity (SC) condition showed main effects (**B**) of with/without SC condition (F(1,104) = 90.73, p = 0.000), (**C**) an interaction effect (F(1,104) = 10.72, p = 0.001) between arousal condition and anterior/posterior condition and (**D**) an interaction effect (F(1,104) = 6.64, p = 0.011) between arousal condition and with/without SC condition. (**E**) Three-way repeated measures ANOVA for arousal condition, inter/intra modular connectivity condition and with/without SC condition showed a main effect of (**F**) inter/intra modular condition (F(1,104) = 17.31, p = 0.000), (**G**) and interaction effect (F(1,104) = 7.12, p = 0.009) between arousal condition and with/without SC condition. (**H**) an interaction effect between inter/intra modular connectivity condition and with/without SC condition (F(1,104) = 7.22, p = 0.008)

**Figure 3:**
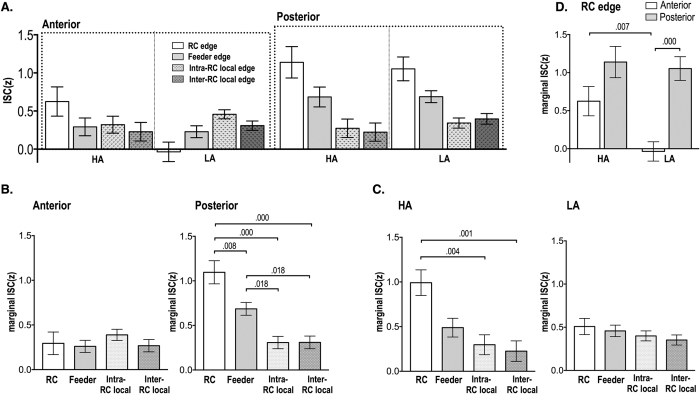
Statistical results for inter-subject correlation (ISC) of functional connectivity in the rich-club organization. The anterior/posterior rich-club edges were determined by the location of rich-club nodes in the anterior or posterior brain regions. (**A**) Three way repeated measures ANOVA for rich-club edge type condition, anterior/posterior region condition and arousal condition showed significant main effects of regions and rich-club edge types. (**B**) The interaction effect between region and edge type conditions (F(3,312) = 14.84, p = 0.000) and the rich-club edge type effect was significant in the posterior region (F(4,416) = 17.66, p < 0.001). (**C**) A significant interaction effect was found between arousal condition and rich-club edge type condition (F(3,312) = 14.84, p < 0.001) showing the edge type effect only in the high arousal (F(4,416) = 6.06, p < 0.001). Two way repeated measures ANOVA of rich-club edges for arousal level condition and anterior/posterior region condition showed significant main effects of arousal condition (F(1,104) = 4.98, p = 0.028) and anterior/posterior condition (F(1,104) = 18.32, p < 0.001).

Statistical results for inter-subject synchrony of functional connectivity according to different edge types are summarized in Table 1 and Figs 2 and 3.

Three-way repeated measures ANOVA for the arousal condition, anterior/posterior brain condition and with/without structural connectivity condition revealed a main effect for the anterior/posterior condition (F(1,104) = 80.73, p < 0.001), demonstrating that the ISC of edges in the posterior brain was higher than in the anterior regions (Fig. 2A and B). The interaction between arousal level and anterior/posterior condition was also significant (F(1,104) = 6.64, p = 0.011) (Fig. 2C). The difference between posterior-anterior ISC was higher in the low arousal state than the high arousal state. The interaction between arousal condition and structural connectivity (F(1,104) = 10.72, p = 0.001) was significant (Fig. 2D). Three-way repeated measures ANOVA for the inter/intra modular connectivity condition, with/without structural connectivity condition and arousal condition (Fig. 2E) showed a main effect for the inter/intra modular edge condition (F(1,104) = 17.31, p < 0.001) (Fig. 2F) and an interaction between inter/intra modular edges and with/without structural connectivity (F(1,104) = 7.22, p = 0.008) (Fig. 2G) and between the arousal condition and the with/without structural connectivity condition (F(1,104) = 7.12, p = 0.009) (Fig. 2H).

The intra-modular edges demonstrated more ISC than inter-modular edges and were significantly more affected by the existence of underlying structural connectivity than were the inter-modular edges.

Three way repeated measured ANOVA for the different rich club edge types condition (RC/feeder/intra-RC local/inter-RC local edges), anterior/posterior region condition and arousal condition (Fig. 3A) revealed a main effect for the anterior/posterior region condition (F(1,104) = 28.66, p < 0.001, demonstrating that the ISC of edges in the posterior brain was higher. A main effect of rich club edge type (F(3,312) = 19.83, p < 0.001) and an interaction between edge types and regions (F(3,312) = 14.84, p < 0.001) were significant. There was no significant difference between rich-club edge types in the anterior brain, but RC edges had higher ISC than feeder and local edges in the posterior brain (F(4,416) = 17.66, p < 0.001) (Fig. 3B). The interaction between edge type and arousal was significant (F(3,312) = 7.89, p < 0.001). Rich club edges were highly synchronized across subjects compared to the other edge types, but only in the high arousal state (F(4,416) = 6.06, p < 0.001) (Fig. 3C).

A two way repeated measures ANOVA of RC edges by arousal condition and by anterior/posterior condition revealed a main effect of the arousal condition on RC edges (F(1,104) = 4.98, p = 0.028), demonstrating greater inter-subject synchrony of RC edges in the high arousal state than the low arousal state. A significant main effect of the region condition on RC edges (F(1,104) = 18.32, p < 0.001) also showed higher ISC of RC edges in the posterior brain than in the anterior brain.

### Inter-subject similarity of edge involvement patterns (ISS)

**Figure 4:**
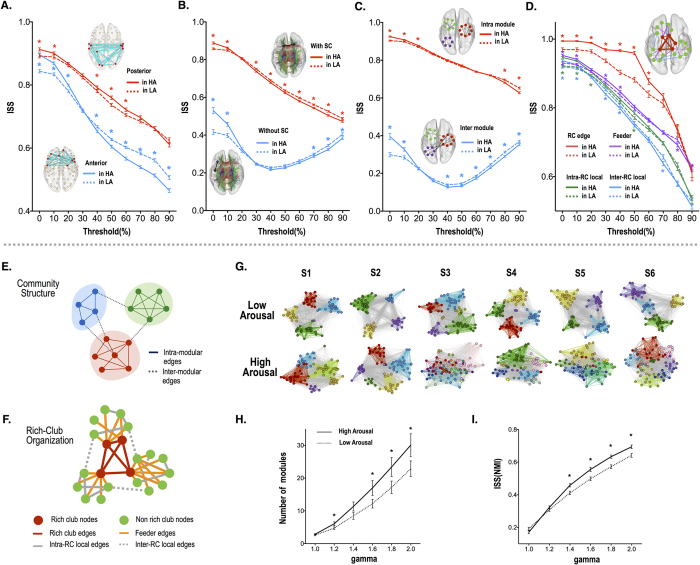
The inter-subject similarity (ISS) of subnetwork patterns with various network thresholds (0–90%) with respect to arousal level and subnetworks of (**A**) anterior/posterior connectivity, (**B**) with/without structural connectivity (SC), (**C**) inter/intra-modular connectivity, (**D**) rich-club(RC)/feeder/intra-RC local/inter-RC local edges in the rich-club organization. *indicates significant difference between low arousal (LA) and high arousal (HA) (p < 0.05, Bonferroni corrected). (**E**) The community structure for modules in Fig. 1C is composed of strong ties which link nodes in the same module (intra-modular edges) and weak ties which connect nodes in different modules (inter-modular edges). (**F**) Rich-club nodes are interconnected with each other by rich club edges. Rich club nodes and non-rich club nodes (or feeder nodes) are connected with feeder edges. Edges that do not connect with rich club nodes are local edges. Local edges are further subdivided into the intra-RC local edges which link feeder nodes in a rich club module and inter-RC local edges which link feeder nodes in different rich club modules. Functional modular structures depending on the arousal level. Exemplary display of modular structures in 6 subjects (S1~S6) at high and low arousal states after modularity optimization are displayed in (**G**). (**H**) The number of modules and (I) inter-subject similarity (ISS) of modular patterns within the whole brain was evaluated using normalized mutual information (NMI) with modularity resolution parameter gamma values from 1 to 2.0. Continuous and dotted lines indicate high and low arousal level. *indicates significant difference according to the arousal levels (paired t-test, p < 0.05, Bonferroni corrected).

The ISS of subnetwork architecture was determined by the adjusted rand index (ARI)^30^, a measure of the degree to which spatial patterns overlap. This analysis indicated that the ISS of subnetwork architecture depends on network thresholds. High thresholds indicate that networks are composed primarily of strong functional connections while lower thresholds indicate network composition includes both weak and strong functional connections. A repeated measures ANOVA showed main effects for the brain region and arousal conditions (Supplementary Table 3 and Fig. 4A). The ISS was higher in the posterior area than the anterior brain for all network thresholds (Fig. 4A). In the posterior brain, the ISS during the high arousal state was significantly higher than during the low arousal state, particularly for network thresholds of 0, 10, 40~60% (paired t-test, p < 0.05). Whereas, the ISS of the anterior edges during the high arousal state was significantly higher than in the low arousal state for network thresholds of 0, 10 and 20%, but this effect was reversed with network thresholds from 40~90% (Fig. 4A).

A repeated measures ANOVA for the ISS of subnetwork patterns (ARI) showed main effects for the presence or absence of underlying structural connectivity and arousal level (Fig. 4B). Connections with corresponding structural pathways had higher ISS than those connections without underlying structural connectivity, regardless of the network threshold (F > 500, p < 0.001). The ISS of the connectivity patterns in the high arousal state was significantly higher than in the low arousal state when networks were constructed with the low thresholds of 0, 10% but reversed with high network thresholds (30–90%).

A repeated measures ANOVA demonstrated main effects of edge type (intra- *versus* inter-modular) and arousal level on the ISS of the functional connectivity patterns (ARI) (Supplementary Table 4 and Fig. 4C). The intra-modular connectivity had higher ISS than inter-modular connectivity, regardless of the network threshold (F > 1000, p < 0.001). The ISS of the functional connectivity patterns in the high arousal state was significantly higher than in the low arousal state with low network thresholds (0~20%) but reversed with high network thresholds (40 ~ 90%). The post-hoc *t*-test showed significantly higher ISS during the high arousal state than during the low arousal state with network thresholds of 10% and 20% for the intra-modular edges and with thresholds of 0 and 10% for the inter-modular edges, but reversed with thresholds 80 ~ 90% and 40~90% for the two edge types respectively (p < 0.05).

Repeated measures ANOVA of ISS showed main effects for rich-club edge types and arousal levels (Supplementary Table 5 and Fig. 4D). The ISS of the RC edges was significantly higher than those of feeder, intra-RC local and inter-RC local edges for most network thresholds (Supplementary Table 6A and B). In most cases, the ISS of edges are higher at the high arousal state than low arousal state, except for the inter-RC local edges.

### Inter-subject similarity of modular patterns of functional networks

The number of modular structures was higher in the high arousal state than in the low arousal state, for most resolution parameters (Fig. 4H). The ISS of modular patterns in the high arousal state was higher than in the low arousal state except for very low resolution (g = 1.0 and 1.2) (Fig. 4I).

## Discussion

Studies of resting-state and task-free networks have implied that large-scale brain networks encode individual characteristics such as maturity^7^, character^31^, finger printing^32^ and task performance^33^. Individual differences with regard to connectivity^19^, cognitive function^34^, cognition^35^ and clinical symptoms^36^ have also been explored.

In this study, we further investigated the manifestations of individual variability by assessing functional networks while subjects engaged in the same series of perceptual tasks that, according to previous studies^37^,^38^,^39^, demand dynamic reconfiguration of brain networks. How inter-individual variability in perceiving, feeling and performing a perceptual task is embedded in the whole brain network was examined with respect to inter-subject synchrony (ISC) of dynamic functional connectivity and inter-subject similarity (ISS) of connectivity patterns during high and low arousal levels in four contrasting divisions of network edges: edges within the anterior *versus* posterior part of the brain, with *versus* without structural connectivity, inter- *versus* intra-module and rich-club (strong) edges (and feeders) *versus* local (weak) edges.

The results of the current study can be summarized as follows: (1) greater inter-individual functional connectivity variability exists within high level cognitive regions compared to sensory brain regions, (2) deviations from structural connectivity contributes to inter-individual variability, (3) inter-modular connectivity encodes more inter-individual variability than intra-modular connectivity, (4) rich-club edges display similar dynamics and patterns of edge involvement across subjects for an identical task stream compared to local edges, suggesting weaker ties are largely responsible for inter-subject variability, and (5) arousal level modulates inter-individual differences in the functional network when performing perceptual tasks.

Various studies have implicated the anterior part of the brain, including the frontal lobe and anterior and medial temporal lobe, as a center for individuality^5^,^31^,^40^,^41^,^42^ argues that variability in functional connectivity within the prefrontal cortex reflects individual differences in cognitive flexibility and attentional capabilities. Our findings concur with this assertion; both nodal activity and functional connectivity in the anterior brain demonstrated high inter-individual variation during a perceptual task. The ISC results of nodal (regional) activity in the current study agree with previous studies that illustrated highly synchronous voxel-wise activity across subjects in the sensory cortices during a series of applied stimuli^43^,^44^,^45^. The synchronous responses in the sensory brain areas to the same stimuli indicate high commonality across individuals in processing sensations while watching a movie.

Using connectivity analysis, we further found that anterior functional connectivity, largely responsible for the higher brain functions, encodes individuality more than posterior connectivity. This encoding is reflected in the lower ISC of edge dynamics and lower ISS for patterns of contributing edges in anterior connectivity compared to posterior connectivity. Diverse combinations (patterns) of functional connectivity with variable connectivity strengths across participants in the anterior brain may account for the individualized experience fundamental to watching a movie. This cognitive-level-dependent (or anterior-posterior) gradient of ISC and ISS is consistent with a previous resting state study^19^, which associated high individual differences with variable responsivity in the frontal cortex of the brain. From this analysis, we can conclude that individual variability resides in the anterior brain.

Functional connectivity is constrained by structural connectivity, but not entirely^46^. The divergence of functional connectivity from the underlying structural connectivity has been used as a biomarker for brain diseases^22^,^23^,^24^. This study demonstrates that the divergence of function from the structure in the brain network can be an important feature in characterizing individual differences. Our examinations of both functional connectivity synchrony (ISC) and pattern similarity of functional connectivity (ISS) revealed high inter-subject variability in the edges that lacked well defined structural connections, suggesting that individual variability is expressed more by functional connectivity diverged from strong structural basis among brain regions.

When performing a brain function, similarly functioning nodes may reside in close proximity to each other, creating a module with strongly-tied interactions (edges). This module may then interact globally with other modules in a context-dependent manner, which is the case for topological properties of the structural brain^18^,^27^,^47^,^48^. In this study, we subdivided global integration among modules into two conceptual frameworks of modularity; modules defined within a community structure^26^ and rich-clubs (a hub for a local community or module) in the rich club organization^27^. Rich-club edges, although they interconnect rich-club modules (global integration), are different from the inter-modular edges defined in the community structure, where dense edges congregate together to compose a module and sparse inter-modular edges remain afterwards.

In both network structures, the hierarchical architecture of the brain follows a general property of modular systems, where strong ties (strong interaction) construct modules, while weak ties (weak interaction) bridge the strongly-bound modules to each other. Intra-modular edges in the community structure and possibly feeder edges have strong structural basis and play as “strong ties”. Intra-modular edges exhibit similar dynamics and subnetwork patterns across individuals. Rich-club edges can also be “strong ties,” particularly within the posterior brain due to their strong anatomical basis.

High inter-subject similarity is found in rich-club edges, implying that individuals conduct a similar profile of global integration during an identical stream of tasks. Meanwhile, high inter-subject variability, thus conceived as the manifestation of individuality, originates mainly from the utilization of inter-modular edges in the community structure or local edges in the rich club organization. Asynchronous dynamics of edge strengths and variable patterns of edge involvements were prominent in the edges spanning between modules compared to those edges within a module. In the rich club framework, local edges show low inter-subject synchrony and low pattern similarity across subjects. More specifically, inter-rich club local edges have lower inter-subject similarity than intra-rich club local edges. These edges can be called “weak ties”. The importance of weak ties (inter-modular connectivity), noted in the sociology as, “the strength of weak ties”^29^, may stem from the inherent flexibility built into weak connections, that other whole brain network studies have recognized^49^. In this study, the strength of weak ties generates high inter-subject variability.

The inter-subject similarity of patterns within subnetworks depends on the network threshold (i.e., functional connectivity level) employed to divide active from inactive edges (Fig. 4). For higher network thresholds, only a small number of edges with strong functional connectivity were contained in the subnetwork while low network thresholds tended to include a wide range of edges, from weak to strong functional connectivity. The inter-subject similarity of most subnetworks, except for subnetworks composed of edges without structural connectivity and inter-modular edges, decreased when network thresholds increased. This implies that edge involvement patterns of strong functional connectivity (high network threshold) are diverse across subjects; whereas, these same networks appear similar when they include weak connectivity as well.

The ISS of subnetworks with inter-modular edges and edges without structural connectivity exhibited a complex dependency on the network threshold; ISS decreased until reaching a threshold of 40%, but increased beyond this threshold (Fig. 4B and C). The patterns of subnetworks composed of edges with strong functional connectivity (above 50%) become more similar across subjects. Explaining the significance of the ISS network threshold dependency on these edges is an intriguing area for future research.

As Nummenmaa, *et al*.^10^ reported, arousal levels differentially modulate the variability of local activity in individual brain networks. In this study, a greater number of edges displayed increased synchronization across participants when arousal levels were high compared to a low arousal state. The association of arousal level with synchronized edges across individuals can be further divided into two network systems, i.e., the attentional network and the sensory network.

Increased ISC during the high arousal state was prominent in the edges connecting the parietal lobe, frontal lobe and limbic system (hippocampus, amygdala, posterior cingulate cortex). Nummenmaa, *et al*.^10^ demonstrated positive associations between arousal levels and the ISC of regional activity in both the visual area and dorsal attention network, thereby arguing that attention-related mechanisms are arousal-contingent. The high arousal state induced by watching a series of emotionally charged video clips would presumably recruit limbic, attentional and emotional processing modules, as well as episodic memory processing substrates. During high arousal states, attentional modulation of cognitive functions is reflected in the enhanced synchrony of brain connectivity from the dorsal attention network to higher sensory cortices and the frontal cortex found in this study (Fig. 1H). We also observed that connectivity within limbic circuits (hippocampus, parahippocampus, amygdala and posterior cingulate cortex) is time locked across individuals to a greater extent during the high arousal state than the low arousal state.

In contrast to attentional and limbic edges, the edges projecting from the primary visual and auditory cortices exhibited significantly higher synchronization in the low arousal state than high arousal state. At low arousal levels, individuals may minimally utilize higher perceptual and cognitive systems to watch a movie and thereby may restrict their processing functions to low-level areas, generating predictable responses to the audio-visual features. Without neuromodulatory feedback from higher lever cortical areas, sensory cortical responses become essentially time-locked across individuals.

Nodes with higher synchrony, however, did not necessarily generate synchronized edges during the high arousal state. For example, the fusiform gyrus, an area with higher nodal ISC, showed greater ISC of functional connectivity with the supramarginal gyrus but lower ISC of functional connectivity with the inferior occipital gyrus during the high arousal state compared to the low arousal state. Furthermore, this apparent decreased synchronization of functional connectivity between a highly synchronized node is inconsistent with increased synchronization of nodal activity at high arousal levels, particularly between the auditory and visual systems. For example, the left superior temporal gyrus and middle occipital gyrus showed higher inter-subject nodal synchrony during the high arousal state, but the inter-subject synchrony of functional connectivity between the two nodes is greater during the low arousal state compared to the high arousal state. These results suggest that previous studies examining inter-individual differences by simply exploring the synchrony of nodal activity would not reveal the defining aspect of individual variability. Instead, synchronized interactions *between* brain regions must be investigated to unravel the elements of individual variability, or differences in individual aspects of subjective perceptual experiences.

At the subnetwork level, arousal states affect the average ISC of edges differentially when comparing edges with, versus without, corresponding structural connectivity and edges in anterior, versus posterior, brain regions. Increased arousal levels strengthen the ISC of the edges within the anterior brain and the edges without structural connectivity but this pattern reverses in the posterior portion of the brain and for the edges with structural connectivity; instead, high arousal states diminish edge strength within posterior brain regions and weaken edges with clearly defined structural substrates (Fig. 2C).

Arousal levels modulate ISS patterns as well, but this dependency is also complex and varies according to network construction thresholds and whether weak connectivity is included in the network model (Fig. 4A,B and C). Heightened arousal promotes high inter-individual similarity of the edge patterns with both weak and strong connectivity (weak network threshold) in contrast to the higher similarity of edge patterns with strong connectivity in the low arousal state compared to the high arousal state.

It should be noted that high arousal induces a significant increase of the inter-subject synchrony at rich club edges compared to the low arousal state and enhances the differentiation of inter-subject synchrony of rich club edges from those of local edges (Fig. 3C and D). Since rich-club edges play an essential role in the global integration of distributed subnetworks, differentiated and increased inter-subject synchrony at rich-club edges during a high arousal state imply that subjects follow a similar stream of global integration during the processing of arousing scenes. This was also evidenced in the inter-subject pattern similarity of edge involvements (or subnetwork) measured using ISS (Fig. 4D). Regardless of arousal levels, the subnetworks with rich-club edges exhibit the highest pattern similarity across subjects, followed by those with feeder edges, intra-rich club local and inter-rich club local edges. Although high arousal induces increased subnetwork pattern similarity across subjects in feeder edges and intra-rich club local edges, the arousal level effect was prominent in subnetwork patterns with rich-club edges. Note that inter-subject pattern similarity of subnetworks with weak ties (inter-modular edges or inter-rich club locals) are lower than those with strong ties (intra-modular or rich-club edges), implicating the strength of the weak ties in the characterization of individual differences.

Arousal modulates individual variability not only in the node, edge and subnetwork patterns but also with respect to the modularity of the brain. A greater number of functional sub-modules emerged during the high arousal state than the low arousal state. This result indicates that the high arousal state promotes a whole brain reorganization towards more refined processing as reflected by the greater differentiation of functional subnetworks. Furthermore, the finely differentiated functional subdivisions (modular patterns) were more similar across subjects during high arousal than low arousal.

In summary, this study was the first to evaluate how individual variability (in the cognitive processing involved in perceiving a series of video clips) manifests in the brain network through inter-individual variation of “functional connectivity” and “functional modular architectures”. The evaluation was performed with respect to temporal synchrony of the nodal activity and edge dynamics, pattern similarity of subnetworks, and modularity in the whole brain network during high arousal and low arousal epochs. Individual differences mainly exist in the connectivity between regions responsible for higher-level cognitive processing and in the connectivity without adhering to structural pathways. Inter-modular connectivity in the community structure and local connectivity in the rich club organization had high variations across individuals in both edge dynamics and patterns of edge involvement. We also showed that arousal diversely modulates the individual variability of edge synchrony, edge patterns and the modularity of the whole brain networks. The strength of weak ties is clear in the emergence of individual variation. Although weak ties are not based on strong structural connectivity, they are strong in differentiating oneself from others. In conclusion, individual variability, particularly differences in individualized perceptual experiences, may reside in the variable and flexible connectivity contained within the large-scale brain network.

## Material and Methods

### Subjects

This study included 15 healthy, right-handed participants (9 males and 6 females, mean age: 25.6 ± 2.82 years). None of the participants had a history of neurological illness or psychiatric disorders. This study followed the human subject guidelines approved by the Institutional Review Board of Severance Hospital, Yonsei University College of Medicine and all participants provided informed consent before the experiment.

### Stimuli presentation and fMRI scanning

**Figure 5:**
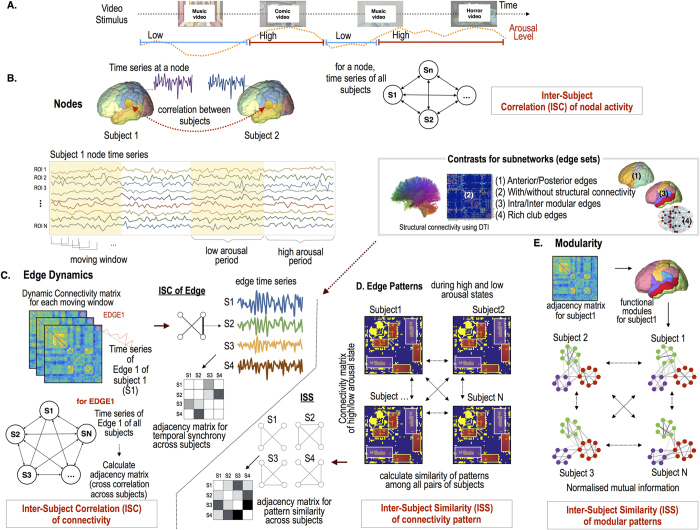
Experimental and analysis procedures. (**A**) Natural movie stimulus composed of 4 types of video clips. Based on the average arousal level of all participants, arousal states were divided into high and low. (**B**) inter-subject correlation (ISC) of nodal activity was defined by average Pearson correlation coefficient between the time series of a region in pairs of participants. (**C**) ISC of dynamic functional connectivity and inter-subject similarity (ISS) of connectivity pattern among the participants. Dynamic functional connectivity was calculated using correlation coefficients of the time series at each sliding window. The ISC of edges, or dynamic functional connectivity, was evaluated by averaging the correlation coefficients (adjacency matrix) of the dynamic functional connectivity (between two nodes) across individuals. The ISS of connectivity patterns was evaluated using the Adjusted Rand Index (ARI) for binarized functional networks across individuals at each arousal state. These measures were evaluated with respect to anterior/poster areas of the brain, with/without structural connectivity, inter/intra-modular edges and edges in the rich-club organization. (**D**) The ISS of the modular structure of functional networks was evaluated using normalized mutual information of modules after the application of modularity optimization.

We presented a set of popular video clips to all participants during fMRI scanning. The stimuli consisted of video clips from 4 different genres: a dance singer’s music video (0 s ~1 min 41 s), a sad movie (1 min 41 s ~6 min 13 s), a singer’s music video “Gang-nam style” (6 min 13 s ~7 min 13 s) and a horror movie (7 min 13 s ~12 min 13 s) for a total duration of 12 min 14 s (Fig. 5A).

### Data acquisition

All participants underwent fMRI scanning with a 3.0 Tesla MRI scanner (Achieva; Philips Medical System, Best, The Netherlands) to obtain T2* weight single shot echo planar imaging (EPI) sequences. Each participant was axially scanned with four dummy scans using the following parameters: 30 ms TE, 2000 ms TR, 90° flip angle, 3.5 mm slice thickness, 0.5 mm slice gap, 36 slices acquired in an ascending interleaved sequence, 80 × 80 matrix, 220 × 220 mm field of view, and a 2.75 × 2.75 × 3.5 mm voxel unit and 0.5 mm slice gap. During presentation of the video clips, a total of 367 scans were acquired. The first five scans were discarded during subsequent preprocessing to eliminate possible MRI transient effects.

We also obtained a high-resolution T1-weighted MRI volume dataset for each subject using a 3D T1-TFE sequence configured with the following acquisition parameters: axial acquisition with a 256 × 256 matrix, 220 mm field of view, 0.86 × 0.86 × 1.2 mm voxel unit, 4.6 ms TE, 9.6 ms TR, 8° flip angle, and 0 mm slice gap.

Diffusion tensor images were obtained using single-shot echo-planar acquisition from 45 non-collinear, non-coplanar diffusion encoded gradient directions with the following parameters: 128 × 128 acquisition matrix with 70 slices, 220-mm field of view, 1.72 × 1.72 × 2 mm^3^ voxels, TE 60 ms, TR 7.9 sec, b-factor of 600 s/mm^2^, without cardiac gating.

### Post-hoc rating of arousal levels

We evaluated arousal levels based on Nummenmaa, *et al*.^10^. After scanning, all participants watched the same movie again outside the MRI scanner and reported their subjective levels of arousal on a 10-point scale. Participants reported their levels of arousal every 30 s, guided by a regular beep tone while watching the film. The low and high arousal states were divided according to the mean arousal score, resulting in 169 and 193 scans, respectively.

### Construction of functional networks

Figure 5 summarizes all the evaluation processes conducted in this study.

To construct networks, we used a parcellation map based on Automated Anatomical Labeling (AAL) map^50^, which partitioned a whole brain into 116 regions. We excluded the cerebellum and used the 92 regions (90 AAL cortical and subcortical regions plus the left and right nucleus accumbens regions, which we manually added to the label map) for network analysis. Preprocessing of fMRI data was conducted using statistical parametric mapping (SPM12, http://www.fil.ion.ucl.ac.uk/spm/, Wellcome Trust Centre for Neuroimaging, London, UK)^51^. All EPI data underwent standard preprocessing steps, including correction of acquisition time delays between different slices, correction for head motion by linearly realigning all consecutive volumes to the first image of the session, and co-registration of T1-weighted images to the first EPI data using the non-linear registration algorithm. Co-registered T1-images was used to spatially normalize functional EPI into MNI template space using nonlinear transformation in SPM12.

fMRI time series for 92 cerebral regions out of the modified AAL map were extracted from the normalized fMRI data in the MNI template space. Time series of eigenvalues corresponding to the first eigenvector, i.e., the mode (derived by applying principal component analysis to time series of voxels in each region), was used as a representative activity for the region (Fig. 1A). The signal fluctuations at each node underwent linear regression analysis to remove rigid motion artifacts (using 12 motion regressors composed of six rigid motion parameters and their derivatives) and global signal changes. The three principal components derived from the signal contained in the white matter and cerebrospinal fluid, followed by high-pass filtering (0.009 Hz), were also used as parameters in the linear regression to remove potential artifacts stemming from these sources. An assessment of individual head motion, using framewise displacement (FD)^52^,^53^ of six motion parameters, demonstrates that the quality of the current data is well within generally acceptable standards in terms of motion artifacts (See more detail about head motion artifacts in the Supplementary Information). To further minimize motion-induced artifacts in the connectivity analysis^52^,^53^, we conducted despiking, a censoring algorithm designed to remove outliers beyond the limit of three standard deviations of the signal^52^. A functional network (adjacency matrix) for a participant was composed of functional connectivity for all pairs of brain regions. Functional connectivity in this study was defined by the Pearson correlation coefficient between the time-series of two brain regions, followed by Fisher’s r-to-z transformation.

### Construction of structural network, modules and rich-clubs

To construct a structural network for an individual, we followed the approach that combines whole brain fiber tractography and structural label on the high-resolution MRI^54^. First, we conducted automated fiber tracking of DTI using DoDTI (Yonsei University, http://neuroimage.yonsei.ac.kr/dodti), with the fourth order Runge-Kutta method. To extract structural networks, whole white matter fiber bundles were reconstructed at approximately 400,000 seed points in the white matter, which was segmented using SPM. The stopping criteria for fiber tracking were a low FA (<0.2) and a rapid change of direction (>60 degree per 1 mm).

In order to construct structural networks, we used 92 cerebral nodes in the individual diffusion tensor space. For this purpose, we co-registered T1-weighted images to DTI using a nonlinear registration algorithm between the T1-weighted images and the non-diffusion-weighted b0-images in DTI for each individual. The AAL map in the template space was transformed into the individual T1-weighted MRI space by applying the inverse nonlinear transformation from individual T1-weighted MRI to the template T1-weighted MRI using the DARTEL toolbox in SPM12^55^. The label map in the individual T1-space was transformed to individual DTI space by applying co-registration function from T1-weighted images to DTI described above.

From whole brain fibers, fiber bundles crossing pairs of regions in the modified AAL map in the individual space were extracted. A structural network (adjacency matrix) for an individual is composed of number of fibers that interconnect every pairs of 92 brain regions in each participant.

The anterior and posterior areas of the brain were defined based on the AAL map including frontal lobe, medial temporal lobe (such as hippocampus, parahippocampal gyrus and amygdala) and temporal pole for the anterior brain and occipital lobe, temporal lobe and parietal lobe for the posterior brain. The cingulate, sensorimotor cortex and subcortical areas were not included in the present study. Since the temporal poles are associated with high-level cognitive processing, we assigned the temporal pole to the anterior brain (Fig. 1B).

From the average structural adjacency matrices of the 15 participants, we divided 92 nodes of the whole brain into 15 modules using the modularity optimization algorithm by Newman^26^. Modularity Q was 0.46 indicating the fraction of the edges that fall within the given groups compared to a null model with random edges (Fig. 1C). We defined the inter/intra-modular edges based on the following modular structure: the intra-modular edge was defined as 2 nodes of the edge existing in a same module and the inter-modular edge between the 2 nodes (Fig. 4E).

We categorized structural edges according to types in the rich-club organization^27^. Rich club nodes (hubs) were identified as nodes with a significant k degree on group-averaging structural connectivity (satisfying the criteria that the fibers were present for at least 50% of the subjects). The edges were then categorized into rich-club (RC) edges, feeder edges, and local edges^27^. We further subdivided local edges into the intra-RC local edges, which link feeder nodes within a module, and inter-RC local edges, which link feeder nodes belonging to different modules (Fig. 4F). For the nodal degree threshold of k > 16, the current network showed significant normalized rich club coefficients *ϕ*_*norm*_(*k*) > 1 (See Supplementary Material) after a permutation testing with 1,000 random networks (P < 0.05, Bonferroni corrected)^27^. The rich club nodes included the bilateral precuneus, hippocampus, putamen, middle occipital cortex, superior frontal cortex, and inferior temporal cortex (Fig. 1D).

### ISC of nodal activity

The inter-subject correlation (ISC) for a node, a measure of brain synchrony between two individuals, was defined as a Fisher’s z-transformed cross-correlation coefficient between the node’s time-series in two participants. We statistically evaluated ISCs for 105 inter-subject pairs (92 nodes for 15 participants during both the high and low arousal states).

### ISC of edge dynamics

The ISC of functional connectivity at a given edge was defined as the average of the Fisher’s z-transformed cross-correlation coefficients of the edge’s time series across all pairs of participants. The edge time series for pairs of regions was calculated at each scan with a window size of 30 scans (60 s) using a sliding window method^56^, which has been demonstrated to reliably detect slow changes in the underlying connectivity from rs-fMRI data^57^.

At each arousal level, we evaluated the ISC of functional connectivity for edges in all subnetworks according to the following four contrasts: (1) brain region (anterior versus posterior); (2) edges with versus without underlying structural connectivity; (3) intra- versus inter- modular as defined by structural network analysis; and, (4) edge type as defined by the rich club schema (RC, Feeder, inter-RC local, intra-RC local).

### ISS of subnetworks

We also evaluated the inter-subject similarity (ISS) of functional connectivity patterns within subnetworks with the above four contrasts. Edges with non-zero functional connectivity (i.e., edges where functional connectivity is higher than a network threshold) within each contrast compose a functional subnetwork; for example, edges having non-zero functional connectivity within the anterior brain or within the posterior brain compose anterior and posterior subnetworks. Network thresholds were defined by order percentile from 0 to 90% of all positive functional connectivity. For example, for a network threshold with 90%, edges with the top 10% of positive functional connectivity within a subregion (e.g., anterior brain) compose a subnetwork. The similarity of functional subnetworks from two subjects was measured using the ARI. The 105 pairwise comparisons of ARI set for the 15 subjects were statistically compared for each contrast.

### ISS of the functional modular structure

To evaluate the individual variability of modular structures of functional networks, we conducted modularity optimization for functional connectivity matrices with various resolution parameters^58^. The resolution parameters γ from 1.0 (low resolution, i.e., small number of modules) to 2.0 (high resolution and many modules) were added to the null model of random edges to control the resolution of the modularity. We compared modular structures of every two subjects using Normalized Mutual Information (NMI)^59^. All functional networks were created after thresholding the connectivity matrix with the false discovery rate (FDR) criterion of FDR < 0.05^60^.

### Statistical analysis of network properties

We compared ISC and ISS results (mostly 105 pairs from 15 subjects) according to contrasts described above using two-way analysis of variance (ANOVA). In the post-hoc analysis of ANOVA, a Bonferroni correction method was used. In the analyses of nodes and edges, an FDR correction for multiple comparisons (FDR < 0.05) was applied to assess the significance level of the nodes and edges in the network.

## Additional Information

**How to cite this article**: Jang, C. *et al*. Individuality manifests in the dynamic reconfiguration of large-scale brain networks during movie viewing. *Sci. Rep.* **7**, 41414; doi: 10.1038/srep41414 (2017).

**Publisher's note:** Springer Nature remains neutral with regard to jurisdictional claims in published maps and institutional affiliations.

## Supplementary Material

Supplementary Information

## Figures and Tables

**Table 1:**
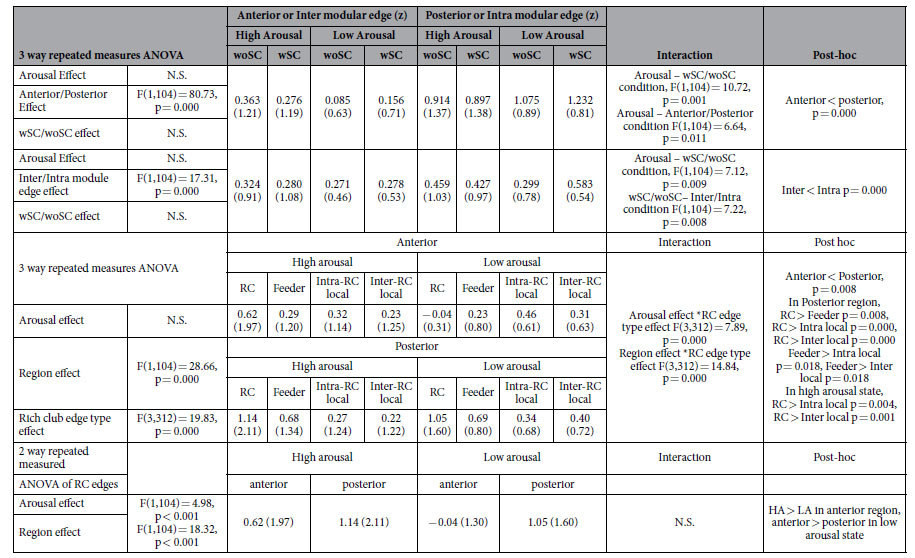
Statistical results of edge ISC for functional connectivity.

^*^N.S. Not Significant. woSC: without structural connectivity, wSC: with structural connectivity. HA: high arousal state, LA: low arousal state. ISC: inter-subject correlation.
