# Supplementary material for: Individuality manifests in the dynamic reconfiguration of large-scale brain networks during movie viewing
Source: Sci Rep. 2017 Jan 23;7:41414. doi: 10.1038/srep41414 (PMC5256084; doi:10.1038/srep41414)
Supplement: Supplementary Information [file srep41414-s1.doc]

**Individuality manifests in the dynamic reconfiguration of large-scale brain networks during movie viewing**

**: Supplementary Information**

Changwon Jang, Elizabeth Quattrocki Knight, Chongwon Pae, Bumhee Park,
 Shin-Ae Yoon, Hae-Jeong Park

**Construction Rich-Club Organization**

We categorized structural edges into three subgroups: 1) inter-modular edges between rich-club nodes (RC edges); 2) intra-modular feeder edges between rich-club nodes and non-rich-club nodes (feeder nodes); and 3) both inter- and intra- modular edges between non-rich-club nodes (local edges) ^1^. We first examined whether the structural networks in our data depict a rich club organization. The rich club coefficient (𝜙), defined below, is used to assess the presence of rich club organizations by comparing the values of an actual network to a random network ^1^. Over a range of degree thresholds, k, the rich club coefficient, 𝜙, is defined as follows:


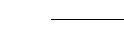


where the rich club coefficient for a given degree threshold,
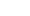
, is the ratio of actual number of edges between remaining nodes,
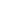
, and the total number of possible edges,
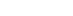
, between them, after removing all nodes with degree less than *k*. Normalized rich club coefficient,
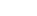
, was calculated as
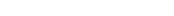
 where
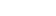
 is the average value of
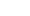
 across 1,000 degree-preserving random networks. The presence of a rich club organization is then determined if
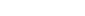
 for any range of k.

The rich club nodes included the bilateral precuneus, hippocampus, putamen, middle occipital cortex, superior frontal cortex, and inferior temporal cortex (Figure 2D). The rich club nodes identified in the present analysis are similar to the rich club nodes described previously (van den Heuvel and Sporn^s 1^ and ^2^ (i.e., the superior frontal gyrus, putamen, insula, hippocampus, precuneus, and precentral gyrus), ^3,4^ (i.e., the insula, middle occipital gyrus, inferior temporal gyrus) and ^5^ (i.e., the anterior/posterior cingulate cortex, midline frontal/posterior, insula, and inferior temporal gyrus).

**Evaluation of Motion Artifacts**

Because serious motion effects are well known confounds in functional connectivity analyses, ^6,7^ we performed despiking, as one of the censoring procedures,^6^ in order to mitigate any motion effects remaining after spatial realignment adjusted for motion induced spatial distortions. To describe the amount of movements in our data quantitatively, we evaluated the quality of the data by calculating framewise displacement (FD) ^6,7^ of six motion parameters for 15 participants at every time point. The ratio of periods with significant artifacts (FD > 1.375 mm, half of the minimal voxel width, 2.75x2.75x4 mm^3^) to whole scan time was less than 4% for all participants examined (mean = 0.76%, ranges = 0 ~ 4% for 15 subjects; 0% for 9 out of 15 subjects). This result is well within generally acceptable limits of subject motion (eg. RMS movement over half a voxel’s width ^7^), suggesting that the quality of our data, with respect to head motion, was satisfactory. We also evaluated the inter-subject correlation (ISC) of FD, which confirmed an absence of synchrony across subjects (r = 0.014, p = 0.44). This result suggests that any residual movement effects would not significantly contribute to the synchrony of the functional connectivity across subjects found in the current study.

**References**

1 van den Heuvel, M. P. & Sporns, O. Rich-club organization of the human connectome. *The Journal of neuroscience : the official journal of the Society for Neuroscience* **31**, 15775-15786, doi:10.1523/JNEUROSCI.3539-11.2011 (2011).

2 van den Heuvel, M. P., Kahn, R. S., Goni, J. & Sporns, O. High-cost, high-capacity backbone for global brain communication. *Proceedings of the National Academy of Sciences of the United States of America* **109**, 11372-11377, doi:10.1073/pnas.1203593109 (2012).

3 Kim, D. J. *et al.* Longer gestation is associated with more efficient brain networks in preadolescent children. *NeuroImage* **100**, 619-627, doi:10.1016/j.neuroimage.2014.06.048 (2014).

4 Kim, D. J. *et al.* Children's intellectual ability is associated with structural network integrity. *NeuroImage* **124**, 550-556, doi:10.1016/j.neuroimage.2015.09.012 (2016).

5 Grayson, D. S. *et al.* Structural and functional rich club organization of the brain in children and adults. *PloS one* **9**, e88297, doi:10.1371/journal.pone.0088297 (2014).

6 Power, J. D. *et al.* Methods to detect, characterize, and remove motion artifact in resting state fMRI. *NeuroImage* **84**, 320-341, doi:10.1016/j.neuroimage.2013.08.048 (2014).

7 Power, J. D., Barnes, K. A., Snyder, A. Z., Schlaggar, B. L. & Petersen, S. E. Spurious but systematic correlations in functional connectivity MRI networks arise from subject motion. *NeuroImage* **59**, 2142-2154, doi:10.1016/j.neuroimage.2011.10.018 (2012).

**Supplementary Table 1. Inter-subject correlation (ISC) of nodal activity (FDR q < 0.05).**

|  | Node | ISC(z) in HA state (mean, std) | | ISC(z) in LA state (mean, std) | | P |
| --- | --- | --- | --- | --- | --- | --- |
| *HA>LA | Left Precentral gyrus | 1.52 | 1.46 | 0.77 | 1.07 | 0.0000 |
|  | Right Precentral gyrus | 1.66 | 1.49 | 0.65 | 1.22 | 0.0000 |
|  | Right Superior frontal dorsal gyrus | 1.21 | 1.63 | 0.75 | 1.30 | 0.0103 |
|  | Right Rolandic operculum | 0.81 | 1.49 | 0.28 | 1.24 | 0.0039 |
|  | Right Superior frontal medial gyrus | 0.89 | 1.41 | 0.27 | 1.41 | 0.0012 |
|  | Left Orbito-frontal medial gyrus | 0.50 | 1.33 | 0.06 | 1.28 | 0.0103 |
|  | Left Posterior cingulum cortex | 1.37 | 1.39 | 0.63 | 1.49 | 0.0002 |
|  | Right Posterior cingulum cortex | 1.52 | 1.36 | 0.73 | 1.21 | 0.0000 |
|  | Left Para Hippocampal gyrus. | 1.96 | 1.32 | 1.35 | 1.31 | 0.0006 |
|  | Right Para Hippocampal gyrus | 2.58 | 1.39 | 1.40 | 1.16 | 0.0000 |
|  | Right Amygdala | 0.70 | 0.95 | 0.16 | 1.06 | 0.0004 |
|  | Right Lingual gyrus | 3.19 | 1.89 | 2.65 | 1.30 | 0.0057 |
|  | Left Occipital superior gyrus | 3.15 | 2.19 | 1.91 | 1.38 | 0.0000 |
|  | Right Occipital superior gyrus | 3.38 | 1.70 | 2.60 | 1.40 | 0.0000 |
|  | Left Occipital middle gyrus | 4.40 | 1.39 | 3.11 | 1.72 | 0.0000 |
|  | Right Occipital middle gyrus | 4.20 | 1.48 | 3.45 | 1.59 | 0.0004 |
|  | Left Fusiform gyrus | 4.99 | 1.45 | 3.62 | 1.52 | 0.0000 |
|  | Right Fusiform gyrus | 5.35 | 1.47 | 3.70 | 1.62 | 0.0000 |
|  | Left Superior parietal gyrus | 3.11 | 1.72 | 1.82 | 1.41 | 0.0000 |
|  | Right Superior parietal gyrus | 3.03 | 1.82 | 1.89 | 1.55 | 0.0000 |
|  | Left Inferior parietal lobule | 0.97 | 1.53 | 0.43 | 1.52 | 0.0036 |
|  | Left SupraMarginal gyrus | 2.30 | 1.75 | 1.10 | 1.24 | 0.0000 |
|  | Right SupraMarginal gyrus | 2.24 | 1.42 | 1.18 | 1.32 | 0.0000 |
|  | Right Precuneus | 2.53 | 1.63 | 1.85 | 1.62 | 0.0004 |
|  | Right Heschl | 2.15 | 1.37 | 1.76 | 1.18 | 0.0033 |
|  | Right Superior temporal gyrus | 3.22 | 1.50 | 2.26 | 1.43 | 0.0000 |
|  | Left Middle temporal gyrus | 2.10 | 1.53 | 1.05 | 1.35 | 0.0000 |
|  | Right Middle temporal gyrus | 3.56 | 1.58 | 1.84 | 1.58 | 0.0000 |
|  | Left TemporalPole middle | 0.56 | 1.13 | 0.10 | 1.14 | 0.0054 |
|  | Right TemporalPole middle | 1.25 | 1.25 | 0.53 | 1.31 | 0.0000 |
| LA>HA | Right Middle frontal gyrus | 0.46 | 1.39 | 0.94 | 1.25 | 0.0072 |
|  | Left Occipital inferior gyrus | 2.84 | 1.75 | 3.92 | 1.76 | 0.0000 |
|  | Right Occipital inferior gyrus | 2.97 | 1.59 | 3.99 | 1.69 | 0.0000 |

- All ISC values are Z values.
* HA : High Arousal state, LA : Low Arousal state.

**Supplementary Table 2. ISC of Edge with LA > HA condition. (FDR q < 0.05)**

| **LA > HA** | | | | | | | | |
| --- | --- | --- | --- | --- | --- | --- | --- | --- |
| **Edge** | **ISC(z) in LA** | **ISC(z) in HA** | **p** |  | **Edge** | **ISC(z) in LA** | **ISC(z) in HA** | **p** |
| IOG.L↔STG.L | 5.32 (4.16) | 0.18 (5.06) | 0.0000 |  | STG.R↔CUN.L | 4.24 (4.91) | 1.21 (4.90) | 0.0000 |
| IOG.L↔FFG.L | 3.32 (5.29) | 0.05 (4.61) | 0.0000 |  | STG.R↔MOG.L | 4.40 (5.55) | 1.08 (4.71) | 0.0000 |
| IOG.L↔FFG.R | 4.25 (4.91) | 0.70 (4.85) | 0.0000 |  | STG.R↔FFG.L | 5.07 (4.09) | 2.55 (5.24) | 0.0005 |
| IOG.L↔IFGtr.R | 2.63 (4.23) | -0.51 (5.28) | 0.0000 |  | STG.R↔IFGop.L | 1.98 (4.63) | -0.57 (5.62) | 0.0007 |
| IOG.L↔CAU.R | 3.10 (4.62) | -0.14 (5.27) | 0.0000 |  | LING.L↔SOG.L | 3.99 (4.70) | 1.13 (6.02) | 0.0004 |
| IOG.L↔IFGop.R | 3.16 (4.13) | 0.19 (4.98) | 0.0000 |  | LING.L↔IOG.R | 2.10 (4.33) | -0.07 (5.25) | 0.0005 |
| IOG.L↔OFGinf.R | 2.52 (5.19) | -0.20 (4.60) | 0.0001 |  | SPG.R↔IPL.R | 2.80 (4.47) | 0.23 (5.54) | 0.0001 |
| IOG.L↔LING.L | 2.68 (4.54) | 0.03 (4.86) | 0.0001 |  | SPG.R↔MFG.L | 3.17 (4.77) | 0.55  (4.81) | 0.0001 |
| IOG.L↔MTG.L | 2.65 (4.95) | 0.13 (4.93) | 0.0002 |  | SPG.R↔IFGtr.L | 1.96 (4.20) | -0.59 (4.85) | 0.0003 |
| STG.L↔FFG.R | 6.72 (4.58) | 2.48 (5.14) | 0.0000 |  | SPG.R↔IFGop.L | 3.19 (4.95) | 0.50 (5.18) | 0.0004 |
| STG.L↔MOG.R | 5.19 (4.12) | 0.69 (5.12) | 0.0000 |  | SPG.R↔MTG.R | 2.75 (4.69) | 0.34 (5.04) | 0.0005 |
| STG.L↔MOG.L | 4.84 (4.98) | 0.98 (4.72) | 0.0000 |  | MTG.L↔ITG.L | 3.76 (4.03) | 0.01 (4.83) | 0.0000 |
| STG.L↔PHG.R | 4.79 (4.37) | 1.86 (5.17) | 0.0000 |  | MTG.L↔OFGinf.R | 2.91 (5.56) | -0.12 (4.55) | 0.0000 |
| STG.L↔SOG.R | 3.18 (4.59) | 0.35 (5.28) | 0.0000 |  | MTG.L↔AMYG.L | 1.98 (4.54) | -0.76 (5.25) | 0.0001 |
| STG.L↔FFG.L | 5.97 (4.81) | 3.74 (4.42) | 0.0003 |  | MTG.L↔TPsup.L | 1.84 (4.48) | -0.41 (4.81) | 0.0005 |
| STG.L↔IOG.R | 3.73 (5.16) | 0.88 (5.67) | 0.0003 |  | MFG.R↔SOG.R | 2.50 (5.17) | -0.07 (5.30) | 0.0005 |
| STG.L↔LING.L | 3.90 (4.28) | 1.50 (4.98) | 0.0005 |  | MFG.R↔PoCG.R | 2.20 (4.66) | 0.03 (4.83) | 0.0012 |
| FFG.R↔HES.R | 5.81 (3.79) | 0.47 (4.24) | 0.0000 |  | CAL.R↔ROL.L | 2.68 (4.75) | -0.13 (4.95) | 0.0000 |
| FFG.R↔STG.R | 6.66 (3.83) | 3.33 (5.21) | 0.0000 |  | CAL.R↔SOG.R | 3.96 (4.80) | 1.31 (4.57) | 0.0001 |
| FFG.R↔MFG.L | 2.59 (4.73) | -0.02 (4.93) | 0.0005 |  | CAL.R↔ITG.L | 3.21 (4.85) | 0.70 (5.40) | 0.0015 |
| FFG.R↔MFG.R | 2.51 (4.80) | 0.02 (4.98) | 0.0006 |  | MTG.R↔SPG.L | 2.31 (5.20) | -0.23 (4.43) | 0.0001 |
| FFG.R↔PRCU.L | 2.62 (5.34) | 0.02 (5.54) | 0.0013 |  | MTG.R↔CUN.L | 3.29 (5.47) | 0.57 (5.55) | 0.0004 |
| MOG.R↔MFG.L | 3.31 (4.43) | 0.08 (4.49) | 0.0000 |  | MTG.R↔PrCG.R | 1.78 (4.50) | -0.46 (5.25) | 0.0011 |
| MOG.R↔STG.R | 4.96 (4.64) | 1.36 (4.87) | 0.0000 |  | ITG.L↔OFGinf.R | 1.99 (4.67) | -0.36 (4.86) | 0.0004 |
| MOG.R↔HES.R | 4.54 (4.25) | 1.46 (4.88) | 0.0000 |  | ITG.L↔CAL.L | 3.44 (4.71) | 0.48 (6.53) | 0.0006 |
| MOG.R↔HES.L | 2.37 (4.01) | -0.34 (5.18) | 0.0001 |  | CUN.L↔IOG.R | 3.05 (4.37) | 0.33 (4.70) | 0.0001 |
| MOG.R↔MFG.R | 2.62 (5.38) | -0.05 (4.72) | 0.0003 |  | PrCG.R↔NAcc.L | 1.27 (5.09) | -0.77 (4.11) | 0.0013 |
| HES.R↔FFG.L | 4.12 (4.01) | 0.98 (5.23) | 0.0000 |  | SPG.L↔PHG.L | 4.25 (4.34) | 1.83 (4.91) | 0.0002 |
| HES.R↔LING.R | 3.23 (4.63) | 0.55 (4.10) | 0.0000 |  | NAcc.L↔TPmid.R | 1.83 (5.28) | -0.33 (4.46) | 0.0016 |
| HES.R↔CAL.R | 2.63 (5.30) | 0.49 (4.73) | 0.0012 |  | SMA.L↔ACC.R | 1.24 (4.93) | -0.51 (6.12) | 0.0018 |
| HES.R↔LING.L | 3.51 (4.12) | 1.60 (4.37) | 0.0017 |  |  |  |  |  |
| **HA > LA** | | | | | | | | |
| **Edge** | **ISC (z) in HA** | **ISC (z) in LA** | **p** |  | **Edge** | **ISC (z) in HA** | **ISC (z) in LA** | **p** |
| AMYG.R↔OFCmid.R | 1.90 (4.06) | -0.54 (4.99) | 0.0004 |  | PRCU.L↔MCC.R | 1.70 (4.70) | -0.43 (5.98) | 0.0018 |
| AMYG.R↔OFCmid.L | 1.87 (4.74) | -0.59 (4.64) | 0.0004 |  | SFGdor.R↔INS.R | 2.58 (4.70) | -0.50 (4.58) | 0.0000 |
| AMYG.R↔STG.L | 2.27 (4.33) | 0.18 (3.95) | 0.0006 |  | SFGdor.R↔PHG.L | 2.16 (4.92) | -0.41 (4.37) | 0.0001 |
| AMYG.R↔IOG.R | 2.66 (4.72) | 0.64 (4.58) | 0.0008 |  | INS.R↔REC.R | 2.16 (4.03) | -0.07 (5.30) | 0.0008 |
| AMYG.R↔IOG.L | 1.98 (3.99) | -0.14 (4.74) | 0.0009 |  | INS.R↔PRCU.R | 2.51 (5.21) | 0.22 (4.11) | 0.001 |
| AMYG.R↔OFGsup.R | 1.85 (3.90) | -0.28 (4.93) | 0.0010 |  | PCC.R↔IFGtr.L | 3.15 (4.84) | -0.71 (5.08) | 0.0000 |
| SMG.L↔PHG.R | 3.64 (5.82) | 0.41 (4.94) | 0.0000 |  | PCC.R↔OFCmid.L | 1.68 (3.77) | -0.41 (4.63) | 0.0004 |
| SMG.L↔FFG.R | 2.77 (5.21) | -0.02 (4.82) | 0.0003 |  | LING.L↔IPL.L | 2.39 (5.57) | -0.43 (5.96) | 0.0007 |
| SMG.L↔ROL.L | 1.94 (4.28) | -0.17 (4.76) | 0.0005 |  | LING.L↔ANG.L | 1.36 (4.77) | -0.73 (4.23) | 0.0008 |
| SMG.L↔PHG.L | 2.80 (5.27) | 0.31 (5.20) | 0.0009 |  | LING.L↔SFGdor.L | 1.86 (5.34) | -0.40 (4.50) | 0.0012 |
| SMG.L↔ANG.R | 1.85 (5.50) | -0.07 (5.71) | 0.0015 |  | FFG.R↔FFG.L | 3.44 (4.68) | 0.40 (4.37) | 0.000 |
| SMG.L↔FFG.L | 2.57 (5.01) | 0.13 (5.11) | 0.0018 |  | FFG.R↔SPG.L | 4.23 (5.25) | 1.33 (5.63) | 0.0002 |
| ROL.R↔PRCU.L | 2.39 (5.06) | -0.54 (4.71) | 0.000 |  | PRCU.R↔TPmid.R | 2.28 (4.26) | -0.06 (4.94) | 0.0003 |
| ROL.R↔PCC.L | 1.97 (4.33) | -0.52 (5.15) | 0.0002 |  | PRCU.R↔IFGtr.L | 2.12 (5.40) | -0.38 (4.54) | 0.0005 |
| ROL.R↔PCC.R | 1.83 (4.64) | -0.55 (4.79) | 0.0003 |  | ROL.L↔PCC.L | 2.39 (4.39) | -0.27 (5.31) | 0.0002 |
| ROL.R↔ANG.R | 2.37 (5.84) | -0.28 (5.55) | 0.0011 |  | HP.L↔TPsup.R | 3.17 (4.75) | 0.68 (4.48) | 0.0002 |
| PHG.R↔SMG.R | 3.19 (4.87) | 0.23 (4.46) | 0.0000 |  | HP.L↔IFGtr.R | 2.80 (5.15) | 0.31 (4.89) | 0.0007 |
| PHG.R↔SFGdor.R | 2.48 (4.57) | 0.15 (3.93) | 0.0002 |  | MOG.R↔SPG.R | 3.42 (5.37) | 0.19 (4.66) | 0.0000 |
| PHG.R↔ITG.L | 3.27 (4.63) | 1.15 (4.88) | 0.0004 |  | MOG.R↔SMG.R | 3.12 (5.56) | 0.17 (4.96) | 0.0003 |
| ANG.R↔PUT.L | 2.23 (4.88) | -0.28 (5.12) | 0.0006 |  | SPG.R↔SFGmed.R | 2.79 (5.48) | -0.01 (5.14) | 0.0008 |
| ANG.R↔PUT.R | 1.99 (5.66) | -0.53 (5.18) | 0.0014 |  | IPL.R↔MFG.R | 2.05 (4.81) | -0.01 (4.67) | 0.0018 |
| PRCU.L↔IPL.R | 1.66 (4.96) | -0.75 (4.89) | 0.0008 |  | INS.L↔AMYG.L | 1.75 (4.61) | -0.39 (4.10) | 0.0005 |
| PRCU.L↔PoCG.R | 1.78 (5.05) | -0.47 (5.08) | 0.001 |  | LING.R↔MTG.R | 2.93 (5.69) | -0.06 (4.72) | 0.0000 |

* Abbreviations for nodes

Anterior cingulate cortex(ACC), Amygdala(AMYG), Angular gyrus(ANG), Caudate(CAU), Calcarine(CAL), Cuneus(CUN), Fusiform gyrus(FFG), Hippocampus(HP), Frontal inferior gyrus opercular parts(IFGop), Frontal inferior gyrus triangular parts(IFGtr), Insula(INS), Parietal inferior lobule(IPL), Temporal inferior gyrus(ITG), Lingual gyrus(LING), Middle cingulate cortex(MCC), Middle occipital gyrus(MOG), Middle Teomporal gyrus(MTG), NucleusAccumbens(NAcc), Frontal inferior orbital gyrus(OFGinf), Olfactory(OLF), Posterior cingulate cortex(PCC), Postcentral gyrus(PoCG), Precentral gyrus(PrCG), Precuneus(PRCU), Putamen(PUT), Parahippocampus(PHP), Rectus(REC), Rolandic operculum(ROL), Superior frontal medial gyrus(SFGmed), Supplementary motor area(SMA), Supramarginal gyrus(SMG), Superior occipital gyrus(SOG), Superior parietal gyrus(SPG), Superior temporal gyrus(STG), Thalamus(THL), Temporal pole Middle(TPmid)

**Supplementary Table 3. Results of two-way repeated measures ANOVA of ISS using ARI for various connectivity thresholds (Anterior/Posterior and high/low arousal condition)**

| Network  Threshold | Degree | Region type effect | | Arousal effect | | Interaction | |
| --- | --- | --- | --- | --- | --- | --- | --- |
|  |  | F | P | F | P | F | P |
| 0 | (1,104) | 157.75 | *0.0000 | 99.55 | *0.0000 | 44.46 | *0.0000 |
| 10 | (1,104) | 216.09 | *0.0000 | 72.01 | *0.0000 | 24.39 | *0.0000 |
| 20 | (1,104) | 341.51 | *0.0000 | 30.81 | *0.0000 | 6.15 | *0.0148 |
| 30 | (1,104) | 419.72 | *0.0000 | 5.95 | *0.0164 | 0.46 | 0.4985 |
| 40 | (1,104) | 385.40 | *0.0000 | 0.32 | 0.5740 | 16.16 | *0.0001 |
| 50 | (1,104) | 366.06 | *0.0000 | 0.00 | 0.9751 | 42.42 | *0.0000 |
| 60 | (1,104) | 356.23 | *0.0000 | 7.30 | *0.0081 | 54.79 | *0.0000 |
| 70 | (1,104) | 374.95 | *0.0000 | 17.40 | *0.0001 | 53.99 | *0.0000 |
| 80 | (1,104) | 303.41 | *0.0000 | 27.47 | *0.0000 | 31.78 | *0.0000 |
| 90 | (1,104) | 272.14 | *0.0000 | 22.09 | *0.0000 | 7.89 | *0.0059 |

**Supplementary Table 4. Results of two-way repeated measures ANOVA of ISS using ARI for various connectivity thresholds (Inter /Intra modular edge and high/low arousal condition)**

| Network  Threshold | Degree | Edge type effect | | Arousal effect | | Interaction | |
| --- | --- | --- | --- | --- | --- | --- | --- |
|  |  | F | P | F | P | F | P |
| 0 | (1,104) | 3415 | *0.0000 | 83.70 | *0.0000 | 51.20 | *0.0000 |
| 10 | (1,104) | 7480 | *0.0000 | 66.06 | *0.0000 | 31.37 | *0.0000 |
| 20 | (1,104) | 22431 | *0.0000 | 3.69 | 0.0573 | 1.60 | 0.2080 |
| 30 | (1,104) | 29748 | *0.0000 | 0.43 | 0.5115 | 12.35 | *0.0007 |
| 40 | (1,104) | 35081 | *0.0000 | 2.33 | 0.1300 | 14.54 | *0.0002 |
| 50 | (1,104) | 26897 | *0.0000 | 1.53 | 0.2186 | 15.90 | *0.0001 |
| 60 | (1,104) | 25980 | *0.0000 | 4.72 | *0.0322 | 7.53 | *0.0071 |
| 70 | (1,104) | 19990 | *0.0000 | 7.24 | *0.0083 | 13.67 | *0.0004 |
| 80 | (1,104) | 10010 | *0.0000 | 24.89 | *0.0000 | 2.20 | 0.1410 |
| 90 | (1,104) | 4064 | *0.0000 | 18.14 | *0.0000 | 2.44 | 0.1213 |

**Supplementary Table 5. Results of two-way repeated measures ANOVA of ISS using ARI for various connectivity thresholds (with/without structural connectivity and high/low arousal condition)**

| Network  Threshold | Degree | SC effect | | Arousal effect | | Interaction | |
| --- | --- | --- | --- | --- | --- | --- | --- |
|  |  | F | P | F | P | F | P |
| 0 | (1,104) | 1719 | *0.0000 | 86.35 | *0.0000 | 79.90 | *0.0000 |
| 10 | (1,104) | 3527 | *0.0000 | 58.96 | *0.0000 | 67.55 | *0.0000 |
| 20 | (1,104) | 12094 | *0.0000 | 1.11 | 0.2940 | 16.52 | *0.0001 |
| 30 | (1,104) | 19713 | *0.0000 | 8.43 | *0.0045 | 8.84 | *0.0037 |
| 40 | (1,104) | 21390 | *0.0000 | 14.35 | *0.0003 | 0.28 | 0.5946 |
| 50 | (1,104) | 16887 | *0.0000 | 10.37 | *0.0017 | 0.10 | 0.7549 |
| 60 | (1,104) | 15469 | *0.0000 | 12.66 | *0.0006 | 6.56 | *0.0119 |
| 70 | (1,104) | 9856 | *0.0000 | 17.60 | *0.0001 | 2.21 | 0.1403 |
| 80 | (1,104) | 3725 | *0.0000 | 30.89 | *0.0000 | 0.00 | 0.9921 |
| 90 | (1,104) | 563 | *0.0000 | 13.53 | *0.0004 | 1.35 | 0.2487 |

**Supplementary Table 6A. Results of two-way repeated measures ANOVA of ISS using ARI for various connectivity thresholds (edges of Rich club organization and high/low arousal condition)**

| Network  Threshold | Degree | RCedge effect | | Degree | Arousal effect | | Interaction | |
| --- | --- | --- | --- | --- | --- | --- | --- | --- |
|  |  | F | P |  | F | P | F | P |
| 0 | (3,312) | 162 | *0.0000 | (1,104) | 131.85 | *0.0000 | 1.38 | 0.2495 |
| 10 | (3,312) | 231 | *0.0000 | (1,104) | 83.70 | *0.0000 | 4.25 | *0.0058 |
| 20 | (3,312) | 490 | *0.0000 | (1,104) | 32.25 | *0.0000 | 13.07 | *0.0000 |
| 30 | (3,312) | 431 | *0.0000 | (1,104) | 22.77 | *0.0000 | 25.89 | *0.0000 |
| 40 | (3,312) | 498 | *0.0000 | (1,104) | 38.82 | *0.0000 | 51.32 | *0.0000 |
| 50 | (3,312) | 552 | *0.0000 | (1,104) | 38.72 | *0.0000 | 50.19 | *0.0000 |
| 60 | (3,312) | 254 | *0.0000 | (1,104) | 5.16 | *0.0251 | 8.22 | *0.0000 |
| 70 | (3,312) | 269 | *0.0000 | (1,104) | 0.02 | 0.8793 | 5.20 | *0.0016 |
| 80 | (3,312) | 154 | *0.0000 | (1,104) | 1.50 | 0.2237 | 0.61 | 0.6099 |
| 90 | (3,312) | 40 | *0.0000 | (1,104) | 0.21 | 0.6468 | 0.45 | 0.7160 |

**Supplementary Table 6B. Post-hoc test (Bonferonii corrected)**

| Network  Threshold | Arousal effect | | | | | | | |
| --- | --- | --- | --- | --- | --- | --- | --- | --- |
|  | RC connection | | Feeder | | Intra-RC local edge | | Inter-RC local edge | |
| 0 | HA>LA | 0.0000 | HA>LA | 0.0000 | HA>LA | 0.0000 | HA>LA | 0.0000 |
| 10 | HA>LA | 0.0000 | HA>LA | 0.0000 | HA>LA | 0.0000 | HA>LA | 0.0001 |
| 20 | HA>LA | 0.0000 | HA>LA | 0.0311 | HA>LA | 0.0001 | . | 0.2318 |
| 30 | HA>LA | 0.0000 | HA>LA | 0.0010 | . | 0.3360 | LA>HA | 0.0006 |
| 40 | HA>LA | 0.0000 | HA>LA | 0.0049 | . | 0.1267 | LA>HA | 0.0424 |
| 50 | HA>LA | 0.0000 | . | 0.1184 | HA>LA | 0.0103 | . | 0.5635 |
| 60 | HA>LA | 0.0000 | . | 1.2912 | . | 0.6184 | .. | 0.8953 |
| 70 | . | 0.2263 | . | 2.4835 | . | 2.2181 | LA>HA | 0.0180 |
| 80 | . | 2.1441 | LA>HA | 0.0339 | . | 0.0894 | . | 1.9740 |
| 90 | . | 2.5769 | . | 1.5315 | . | 0.9352 | . | 0.2895 |

| Network Threshold | RC vs Feeder(Fe) | | RC vs Intra-RC local(Lo1) | | RC vs Inter-RC local(Lo2) | | Feeder(Fe) vs Intra-RC local(Lo1) | | Feeder(Fe) vs Inter-RC local(Lo2) | | Intra-RC local(Lo1) vs Inter-RC local(Lo2) | |
| --- | --- | --- | --- | --- | --- | --- | --- | --- | --- | --- | --- | --- |
| 0 | RC>Fe | 0.0000 | RC>Lo1 | 0.0000 | RC>Lo2 | 0.0000 | . | 0.1466 | . | 0.5651 | . | 2.0163 |
| 10 | RC>Fe | 0.0000 | RC>Lo1 | 0.0000 | RC>Lo2 | 0.0000 | . | 1.4013 | Fe>Lo2 | 0.0160 | . | 2.8218 |
| 20 | RC>Fe | 0.0000 | RC>Lo1 | 0.0000 | RC>Lo2 | 0.0000 | . | 0.5093 | Fe>Lo2 | 0.0000 | Lo1>Lo2 | 0.0022 |
| 30 | RC>Fe | 0.0000 | RC>Lo1 | 0.0000 | RC>Lo2 | 0.0000 | Fe>Lo1 | 0.0000 | Fe>Lo2 | 0.0000 | Lo1>Lo2 | 0.0000 |
| 40 | RC>Fe | 0.0000 | RC>Lo1 | 0.0000 | RC>Lo2 | 0.0000 | Fe>Lo1 | 0.0000 | Fe>Lo2 | 0.0000 | Lo1>Lo2 | 0.0000 |
| 50 | RC>Fe | 0.0000 | RC>Lo1 | 0.0000 | RC>Lo2 | 0.0000 | Fe>Lo1 | 0.0011 | Fe>Lo2 | 0.0000 | Lo1>Lo2 | 0.0000 |
| 60 | RC>Fe | 0.0000 | RC>Lo1 | 0.0000 | RC>Lo2 | 0.0000 | Fe>Lo1 | 0.0000 | Fe>Lo2 | 0.0000 | Lo1>Lo2 | 0.0000 |
| 70 | RC>Fe | 0.0000 | RC>Lo1 | 0.0000 | RC>Lo2 | 0.0000 | Fe>Lo1 | 0.0000 | Fe>Lo2 | 0.0000 | Lo1>Lo2 | 0.0000 |
| 80 | RC>Fe | 0.0000 | RC>Lo1 | 0.0000 | RC>Lo2 | 0.0000 | Fe>Lo1 | 0.0000 | Fe>Lo2 | 0.0000 | Lo1>Lo2 | 0.0000 |
| 90 | . | 3.0552 | RC>Lo1 | 0.0000 | RC>Lo2 | 0.0000 | Fe>Lo1 | 0.0000 | Fe>Lo2 | 0.0000 | . | 0.0523 |

**Supplementary Table 7**. The number of module and the similarity(ISS) of modular pattern in high and low arousal state with the various gammas.

| Gamma | N of modules | | P | NMI of modules | | P |
| --- | --- | --- | --- | --- | --- | --- |
|  | High arousal | Low arousal |  | High arousal | Low arousal |  |
| 1.0 | 2.87(0.74) | 2.60(0.83) | 0.3343 | 0.17(0.10) | 0.19(0.09) | 0.0684 |
| 1.2 | 6.07(2.28) | 4.73(1.58) | *0.0293 | 0.32(0.09) | 0.31(0.10) | 0.1748 |
| 1.4 | 11.33(5.35) | 8.47(4.02) | 0.0615 | 0.46(0.10) | 0.41(0.11) | *0.0000 |
| 1.6 | 17.00(8.78) | 12.20(5.19) | *0.0268 | 0.56(0.13) | 0.50(0.12) | *0.0000 |
| 1.8 | 23.40(11.13) | 17.20(7.39) | *0.0243 | 0.63(0.11) | 0.57(0.12) | *0.0000 |
| 2.0 | 30.07(13.37) | 22.87(9.27) | *0.0291 | 0.69(0.11) | 0.64(0.12) | *0.0000 |
